# Supplementary material for: Can ‘Script Elicitation’ Methods Be Used to Promote Physical Activity? An Acceptability Study
Source: Behav Sci (Basel). 2024 Jul 5;14(7):572. doi: 10.3390/bs14070572 (PMC11273430; doi:10.3390/bs14070572)

Can ‘script-elicitation’ methods be used to promote physical activity? An acceptability study

Supplementary Document S1: Baseline interview schedule (including Script Elicitation)

SEMI-STRUCTURED INTERVIEW QUESTIONS

Existing physical activity pattern

1. What does ‘physical activity’ mean to you?

2. Do you consider yourself to be a physically active person? Why/why not?

- Are you conscious of the times when you aren’t very physically active?

3. Do you do much physical activity as part of your current routine? Why/why not?

prompts:

- What activity/activities?
- What intensity?
- For how long have you been doing it?
- Why do you do it?

4. Are there times in your current routine when you are inactive (i.e. don’t move much) for long periods?

Follow-up questions:

- When?
- Why?
- What activity/activities?

Attempts to change physical inactivity patterns

5. Imagine that someone with the exact same daily routine, and physical activity patterns as you, wanted your advice on how to get more active. What would you suggest?

SCRIPT ELICITATION PROCEDURE

Eliciting existing script

6. You mentioned earlier that you would like to introduce more physical activity into your everyday routine. Next, what we’re going to ask you to do is identify a more specific period within your routine for us to focus on in more detail.

   First, we’d like you to outline the period around work when you think it would be easiest to introduce some more physical activity; ***this could be either your time before work OR your lunch break OR your time after work?***

   - *Prompts*: before picking, try to think of any reasons why engaging in more physical activity during either of these periods would be particularly challenging.

7. Please describe this period of your routine in fine detail, using the start and end points that we just established? *(Researcher writes start and end point on post-it notes and places them at either end of the table)*

- ***Give an example to the participant if not clear:*** e.g., if I was describing my bathroom routine, I put toothpaste on the toothbrush, I brush my teeth for 2 minutes, I put the toothbrush away, I wash my face, I dry my face etc.).
- *(As participant lists behaviours/activities, researcher writes each one down on post-it notes and places it on the table in order)*
- *If behaviour described is vague or could be more detailed, ask the participant to expand or break the behaviour down further.*
- *Continue until you feel that each behaviour is fully explained to an appropriate level of detail.*

8. In your head, which of these behaviours do you feel ‘go together’ as part of your routine?

- *If unclear:* for example, behaviours might feel like they go together because you always do them together, or because you do them in the same location.
- (*As participant lists behaviours that go together, researcher moves these behaviours into a small vertical ‘cluster’ on the table to show that they go together*)

9. Looking at this routine, what would you say prompts you to move from one of these actions (or group of actions) to the next? You may have touched on some of these prompts when you were describing your routine, which can include specific time points, previous task completion or arriving at/leaving a specific location/environment.

- (*Based on the prompts that the participant gives, researcher writes these down on a different coloured post-it note and places this before the behaviour it prompts).*

10. Is the routine shown on the table in front of you accurate?

(Researcher takes picture of current routine for future reference)

Designing new, alternative script

11. Looking at your current routine that we have just described, is there anything that you think we could change to try and add a little bit more physical activity into your routine or reduce the time spent sitting?
    - This could be done by removing behaviours, adding new ones and/or reorganizing existing ones.
    - *If struggling to think of suggestions, guide the participant to potential periods to focus on:* maybe we could start by looking at periods where you currently aren’t doing any physical activity or are sitting for a prolonged period? How could we change or reorganize that to introduce (even a small amount) of physical activity or reduce the time you spend sitting?
    - *(When participant suggests change, researcher makes appropriate changes to routine by taking away post-it notes, reorganizing them or adding new behaviours on a different coloured post-it notes [based on type of suggested change]).*
    - Based on this/these change(s), I’m going to create some small plan/reminder sentences to help prompt you to follow this new routine. For this new part, would it be appropriate for me to add ‘When I X, I will Y’.
    - (*Researcher proposes prompts/plans to act as reminders based on identified cues e.g., when I finish my emails, I will go on a 15-minute walk – researcher should write these on a post-it note and place on the table, above the new behaviour/reorganized behaviour/where old behaviour was)*

(Researcher takes a picture of new routine for future reference)

Thank you for your time, I will now stop the recording. After this meeting, I am going to format this new routine into a flow chart, which will include your prompts/reminders. I will email this to you later today, and we’d like for you to try to adhere to this routine over the next 7 days. It might be helpful to print off the flow chart and place it somewhere prominent in your routine to help remind you, for example on your bedroom door. Thank you!

Supplementary Document S2: Follow-up interview schedule

SEMI-STRUCTURED INTERVIEW QUESTIONS

Reflections on attempts to adhere to new script

1. How did you find seeing your routine displayed in front of you like we did last week?

- *Possible follow-up:* Do you think it was helpful? Why/why not?

2. How did you get on with trying to change your routine since last week?

Possible prompt:

- Were you able to make the changes that you planned to make?
- Was there anything you think that we could have done to make it easier for you to implement these changes?

3. Did you notice any changes to your physical activity/sitting?

- If you were talking to a friend about what we did last week, what benefits would you describe now that you’ve had a week to give the new routine a go?

4. Did you come across any barriers to making the changes you planned to make?

- If so, did you manage to overcome them?
    a. If yes, how?
    b. If no, why not?

5. Do you think it was beneficial to identify a specific part of your routine that we could target?

- Is there anything that you think we could have done for this part of the activity to make it easier for you to identify a specific period to target?

6. How helpful in general was the interview last week to you?

- *Possible prompts:*
    a. What, if anything, did you take from the first interview? (i.e. did you learn anything new?)
    b. Any specific aspects of the first interview?
    c. How useful was the plan that we drew up last week?
    d. Did you use the flowchart that we gave you?

7. Would you recommend taking part to someone with the same routine as you?

- If already active ‘we established last week that you’re already pretty active, do you think this method could be beneficial for people who are already physically active but would like to do more?’

ACCEPTABILITY QUESTIONS

8. How much did you *like or dislike* the method that we used last week, on a scale of 1-5 (1= strongly dislike, 5= strongly like)?
- What aspects did you like/dislike?
- Did you like the use of post-it notes when mapping your routine and making changes?

9. How *comfortable* did you feel engaging with the script elicitation procedure, on a scale of 1-5 (1= very uncomfortable, 5= very comfortable)?
- Were the questions asked to you clear, and was it clear what we wanted you to do?

10. How much *effort* do you feel it took to engage with the script elicitation procedure, on a scale of 1-5 (1= no effort at all, 5= huge effort)?
- Is there anything that we could have done differently that would have made it less effortful?

11. How *confident* did you feel about engaging with the script elicitation procedure, on a scale of 1-5 (1=very unconfident, 5= very confident)?
- What in particular made you feel more/less confident?
- Did giving examples make you feel more confident in picking a specific part of your routine to target?

12. ‘Script elicitation *has improved* my physical activity’ (1=strongly disagree, 5= strongly agree).

13. ‘It's clear to me how script elicitation *will help to improve* my physical activity’ (1=strongly disagree, 5= strongly agree)

14. ‘The script elicitation procedure *interfered* with some of your other priorities’. (1= strongly disagree, 5= strongly agree)
- If so, in what way did it interfere?
- What could we have done differently to prevent this interference?

That is all I have for you today. Are there any questions that you wanted to ask?

Can 'script-elicitation' methods be used to promote physical activity? An acceptability study

Supplementary Document S3: Baseline and follow-up questionnaires

Q1 The questions will ask you about the time you spent being physically active in the last 7 days. Please answer each question even if you do not consider yourself to be an active person. Please think about the activities you do at work, as part of your house and garden work, to get from place to place, and in your spare time for recreation, exercise or sport.

Q4 Think about all the vigorous activities that you did in the last 7 days. Vigorous physical activities refer to activities that take hard physical effort and make you breathe much harder than normal. Think only about those physical activities that you did for at least 10 minutes at a time.

Q1 During the last 7 days, on how many days did you do vigorous physical activities like heavy lifting, digging, aerobics, or fast bicycling?

○ _ days per week (1) ____

○ No vigorous physical activities (2)

Skip To: Q7 If During the last 7 days, on how many days did you do vigorous physical activities like heavy lifti... = No vigorous physical activities

Q2 How much time did you usually spend doing vigorous physical activities on one of those days?

○ _ hours per day (1) ____

○ _ minutes per day (2) ____

○ Don't know/not sure (3)

Q7 Think about all the moderate activities that you did in the last 7 days. Moderate activities refer to activities that take moderate physical effort and make you breathe somewhat harder than normal. Think only about those physical activities that you did for at least 10 minutes at a time.

Q3 During the last 7 days, on how many days did you do moderate physical activities like carrying light loads, bicycling at a regular pace, or doubles tennis? Do not include walking.

○ _ days per week (1) ____

○ No moderate physical activities (2)

Skip To: Q10 If During the last 7 days, on how many days did you do moderate physical activities like carrying li... = No moderate physical activities

Q4 How much time did you usually spend doing moderate physical activities on one of those days?

○ _ hours per day (1) ____

○ _ minutes per day (2) ____

○ Don't know/not sure (3)

Q10 Think about the time you spent walking in the last 7 days. This includes at work and at home, walking to travel from place to place, and any other walking that you have done solely for recreation, sport, exercise, or leisure.

Q5 During the last 7 days, on how many days did you walk for at least 10 minutes at a time?

○ _ days per week (1) ____

○ No walking (2)

Skip To: Q13 If During the last 7 days, on how many days did you walk for at least 10 minutes at a time? = No walking

Q6 How much time did you usually spend walking on one of those days?

○ _ hours per day (1) ____

○ _ minutes per day (2) ____

○ Don't know/not sure (3)

Q13 The last question is about the time you spent sitting on weekdays during the last 7 days. Include time spent at work, at home, while doing course work and during leisure time. This may include time spent sitting at a desk, visiting friends, reading, or sitting or lying down to watch television.

Q7 During the last 7 days, how much time did you spend sitting on a week day?

- ○ _ hours per day (1) ____
- ○ _ minutes per day (2) ____
- ○ Don't know/not sure (3)

Supplementary Figure S1: Example current script (Participant 10)

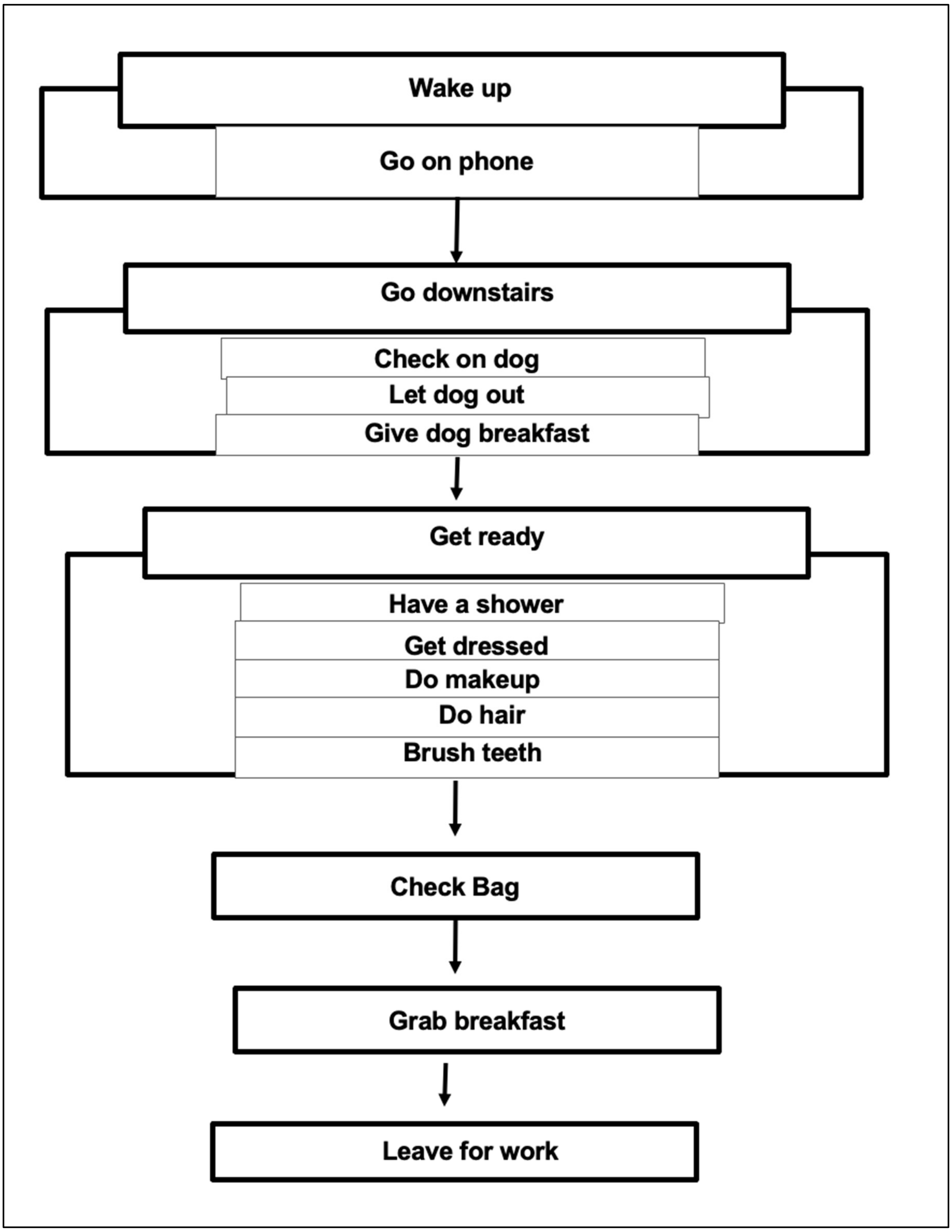

Supplementary Figure S2: Example alternative script (Participant 10)

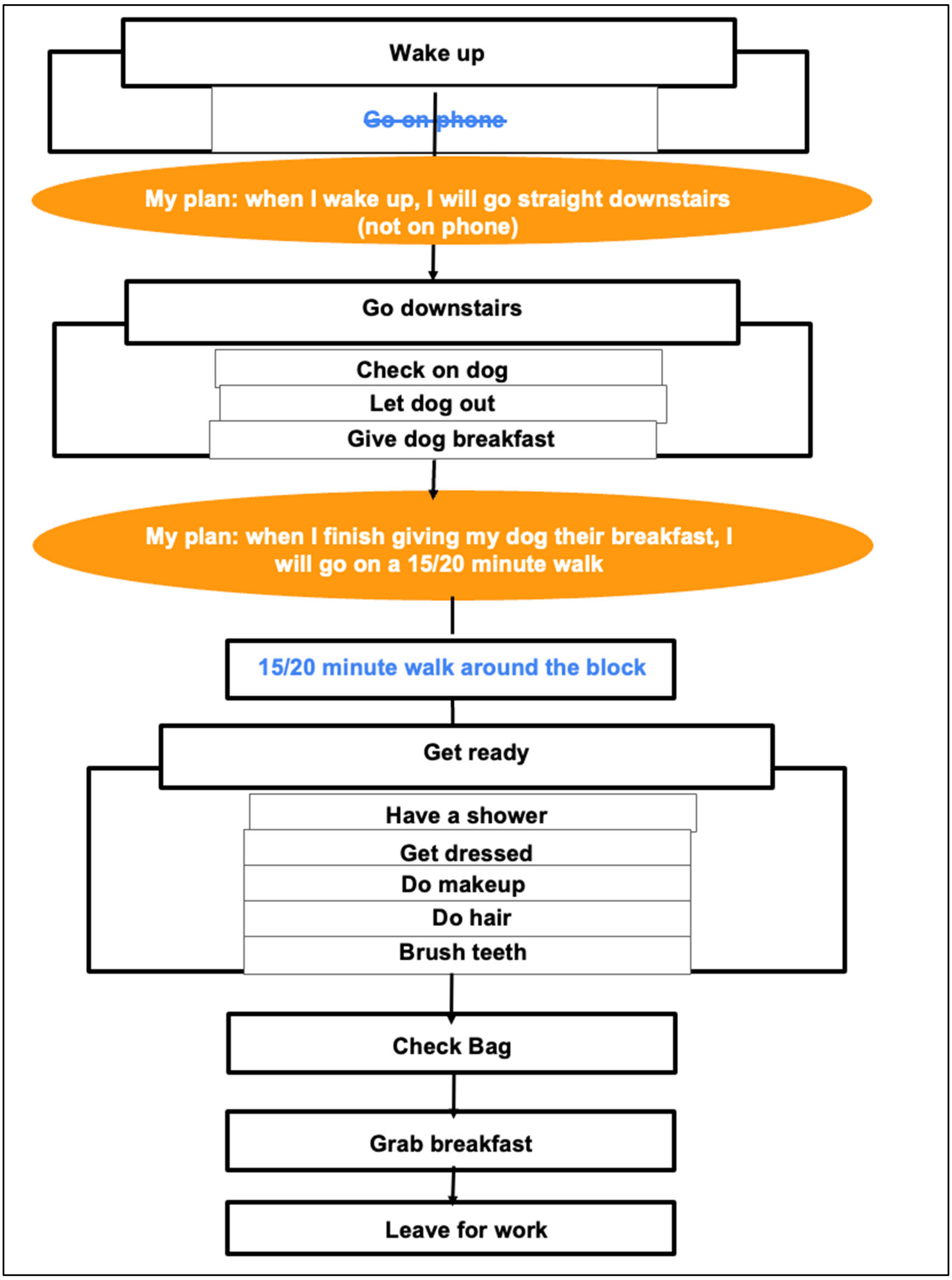

Supplement: Supplementary file 1 [file behavsci-14-00572-s001.zip › behavsci-3072467-supplementary.pdf]
